# Evaluation of a host-protein signature score for differentiating between bacterial and viral infections: real-life evidence from a German tertiary hospital

**DOI:** 10.1007/s15010-024-02384-w

**Published:** 2024-09-09

**Authors:** Laura Wagner, Heike Schneider, Peter B. Luppa, Kathrin Schröder, Nina Wantia, Christiane Querbach, Samuel D. Jeske, Tobias Lahmer, Kathrin Rothe, Miriam Dibos, Florian Voit, Johanna Erber, Christoph D. Spinner, Jochen Schneider, Julian Triebelhorn

**Affiliations:** 1 TUM School of Medicine and Health, Department of Clinical Medicine, Clinical Department for Internal Medicine II, University Medical Center, Technical University of Munich, Ismaninger Str. 22, 81675 Munich, Germany; 2 TUM School of Medicine and Health, Department of Clinical Chemistry and Pathobiochemistry, University Medical Center, Technical University of Munich, Munich, Germany; 3 TUM School of Medicine and Health, Department of Medical Microbiology, Immunology and Hygiene, University Medical Center, Technical University of Munich, Munich, Germany; 4 TUM School of Medicine and Health, Department of Pharmacy, University Medical Center, Technical University of Munich, Munich, Germany; 5 TUM School of Medicine and Health, Department of Virology, University Medical Center, Technical University of Munich/Helmholtz Centre Munich, Munich, Germany

**Keywords:** Antibiotics, Infection, Blood stream infections, Procalcitonin, C-reactive protein

## Abstract

**Purpose:**

A host-protein signature score, consisting of serum-concentrations of C-reactive protein, tumour necrosis factor-related apoptosis-inducing ligand, and interferon gamma-induced protein 10, was validated for distinguishing between bacterial and viral infections as an antimicrobial stewardship measure for routine clinical practice among adult patients in a German tertiary hospital.

**Methods:**

This single-centre, explorative study prospectively assessed the host-protein signature score, comparing it with serum procalcitonin (PCT) in patients with blood stream infections (BSI) and evaluating its efficacy in patients with viral infections against the standard of care (SOC) to assess the need for antibiotics due to suspected bacterial super/coinfection. Manufacturer-specified threshold scores were used to differentiate viral (< 35) and bacterial (> 65) infections.

**Results:**

Ninety-seven patients (BSI [*n* = 56]; viral infections [*n* = 41]) were included. The score (cut-off score > 65) tended to detect BSI with higher sensitivity than did PCT (cut-off > 0.5 ng/mL) (87.5% vs. 76.6%). Three patients (5.4%) with BSI had a score < 35. One patient with BSI did not receive antibiotic treatment following SOC prior to positive blood culture results. Among patients with viral infections, 29 (70.7%) had scores > 65, indicating bacterial superinfections. Additionally, 11 patients (26.8%) had scores < 35, indicating no bacterial superinfections. In total, the antibiotic treatment discrepancy in the viral group between the SOC and a host-protein signature score guided approach was 2/41 patients (4.9%).

**Conclusion:**

The score tended towards a higher sensitivity in detecting BSI than that with PCT. However, its impact on reducing antibiotic use in viral infections was minor compared with that of SOC.

**Supplementary Information:**

The online version contains supplementary material available at 10.1007/s15010-024-02384-w.

## Introduction

Accurate detection of bacterial infections is essential for the prudent and responsible use of antibiotics. This serves a dual purpose: minimising morbidity and mortality and preventing medically unneeded antibiotic therapy. Misdiagnosis related to disease aetiology can lead to antibiotic misuse. Two studies reported rates of unnecessary antibiotic use of approximately 30% [1, 2].

In current clinical practice, diagnosing a bacterial infection primarily relies on clinical assessment and biomarkers [3]. Procalcitonin (PCT) is a biomarker that is frequently used as a decision aid to determine the presence or absence of a bacterial infection. [3–5] However, the sensitivity and specificity of PCT are not sufficient to reliably exclude or confirm a bacterial infection in all clinical infectious settings [3–5]. Thus, distinguishing bacterial from viral infections, particularly with the aid of biomarkers, remains a challenge. Hence, current guidelines for community-acquired pneumonia advise against using biomarkers as a decision aid to differentiate between viral and bacterial aetiologies [3, 6, 7].

To increase the predictive accuracy for bacterial or viral infections, a computational score of multiple host-protein signatures has been introduced by MeMed (Tirat, Carmel, Israel); it is CE-IVD cleared and available in the United States, European Union, and Israel. The score is determined by three variables: the serum concentration of C-reactive protein (CRP), interferon gamma-induced protein 10 (IP-10), and the tumour necrosis factor-related apoptosis-inducing ligand (TRAIL). The ImmunoXpert™ software (MeMed, Tirat Carmel, Israel) uses the results of these variables, measured using chemiluminescent immunoassays (CLIA), to calculate the host-protein signature score, which ranges from 0 to 100. As per the manufacturer’s guidelines, a high score (66–100) was considered indicative of bacterial infection, while a low score (0–34) indicated viral infection. A score of 35–65 indicated that the likelihood of bacterial and viral infections was unclear. [8]

The present study aimed to compare the score with PCT in patients with blood stream infections (BSI). Additionally, the study sought to evaluate the performance of the assay in patients with various viral infections, diagnosed using positive polymerase chain reaction (PCR) against the standard of care (SOC) to assess the need for antibiotics due to suspected bacterial super/coinfection.

## Methods

### Study design

**Fig. 1:**
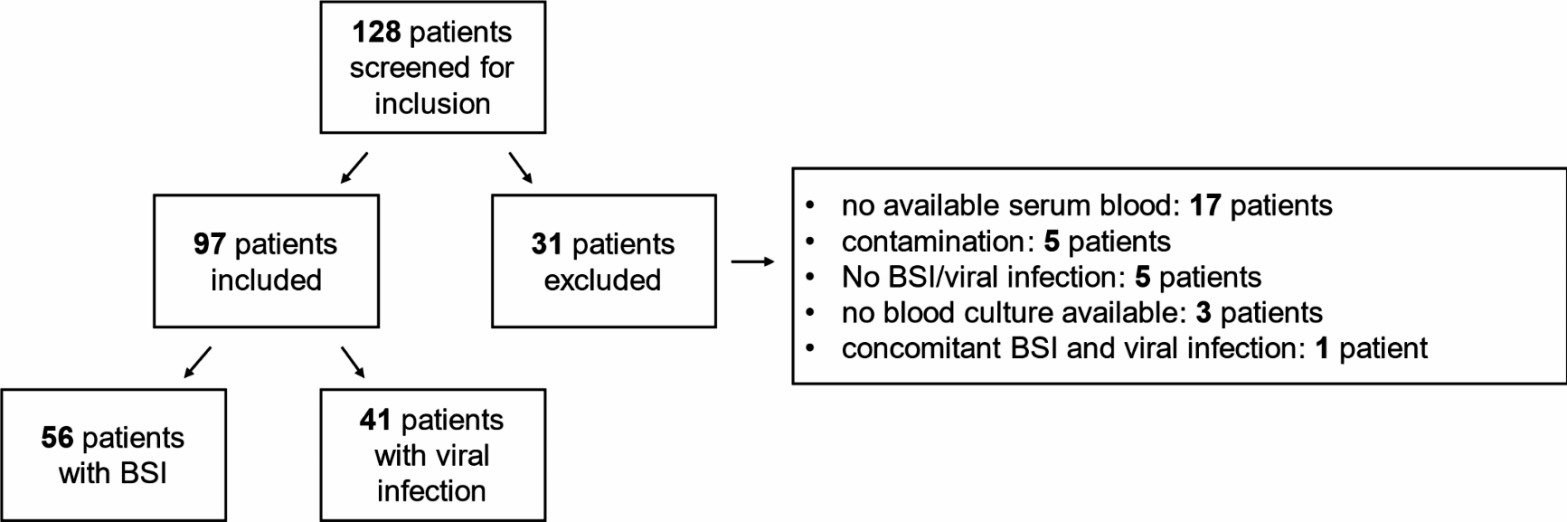
Overview of the study cohort. BSI, blood stream infection

This prospective, monocentric, explorative study was performed at the University Hospital rechts der Isar, Technical University of Munich, in Munich, Germany, between November 2022 and June 2023. Patients were recruited from the emergency department, the general wards of the department of urology, cardiology, gastroenterology, oncology, nephrology as well as the intensive care unit for gastrointestinal and infectious diseases. The general inclusion criteria were as follows: (1) age of ≥ 18 years; (2) the presence of at least one sign of inflammation, defined as a fever of > 38 °C or CRP levels > 5 mg/dL; and (3) diagnosis of confirmed BSI (Group A) or confirmed viral infection (Group B). Viral infection was confirmed using PCR in respiratory material or blood samples and a concurrent negative blood culture result. We excluded all patients without available/sufficient serum samples (the main reason was that the serum was not centrifuged on time for the host-protein signature CLIA), with coagulase-negative staphylococci detected in blood cultures without accompanying evidence of systemic infection, with viral infection but missing blood culture diagnostic results, and with simultaneous BSI and viral infection (Fig. 1).

In case of a suspected infection, indicated by fever or elevated inflammation markers such as CRP, the standard of care procedure included collecting at least one aerobic and one anaerobic blood culture. For suspected viral infections, PCR diagnostic was performed using either respiratory or blood samples to identify the suspected viruses. The decision to initiate antibiotic therapy was based on the likelihood of a bacterial infection, the severity of the disease, the underlying comorbidities, and in accordance with the relevant standard operating procedure (SOP) for that specific infection. In general, the final decision to start or withhold antibiotic treatment was made in consultation with a specialist or senior physician. The selection of antibiotic therapy depended on the type of infection and adhered to local antimicrobial stewardship (AMS) guidelines as outlined in the relevant SOP.

Immunosuppressive therapy was defined as therapy with steroids in any dosage, or immunosuppressive/immunomodulatory agents. Immunocompromised patients were defined as patients receiving immunosuppressive therapy, neutropenic patients, solid-organ or stem cell transplanted patients, and patients with active lymphoma or leukaemia.

The scores were unavailable to the attending physician and, consequently, had no impact on the clinical decision-making process. The aetiology of the infection (bacterial or viral) was not disclosed to the staff who carried out the CLIA.

### Sample collection and selection

The score was calculated in all patients with available serum samples. If PCT had not been determined in the blood sample from which the CLIA was performed, PCT results were collected from blood samples up to 2 days earlier or 2 days later, if available.

The CLIA was performed using serum obtained during routine blood sampling. It was performed after the confirmation of BSI or viral infection. If no serum was available on the same day, it was performed as part of the subsequent routine blood sampling. Samples were frozen at − 20 °C till analysis. CLIA tests for the determination of the score were performed in batches. Only one freeze–thaw cycle was permitted.

### Laboratory analyses

Each blood sample was drawn into a serum separation tube (Sarstedt) and processed by centrifugation at 1,500 x g within 1 h after arrival at the central laboratory. A total of 350 µL of sample material was used for the three score parameters, analysed using the LIAISON^®^ XL CLIA fully-automated analyser (DiaSorin Deutschland GmbH, Dietzenbach, Germany). The device performs the measurement of the three host immune parameters and integrates the respective results into the unique host-protein signature score (LIASON MeMed BV^®^ score). On the LIASION XL device, the CRP measurement is traceable to the standard reference material IFCC/BCR/CAP CRM 474, whereas TRAIL is traceable to the World Health Organization reference reagent TRAIL [human, rDNA, E. coli-derived] NIBSC 04/166. For IP-10, a suitable reference material is not available. Therefore, the measurand is traced to an internal standard characterised by amino-acid analysis. The reproducibility acceptance criterion for TRAIL, IP-10, and CRP of a coefficient of variation (CV) of ≤ 15% was checked by serial measurements of control samples delivered by DiaSorin [8]. The precision data of the immune markers run on the LIAISON XL device were as follows: CV for CRP was 8.0%, for IP-10 5.3%, and for TRAIL 8.5%.

The PCT measurements were run on the routinely used immunoassay analyser cobas e801 (Roche Diagnostics, Mannheim, Germany), according to the manufacturer’s original instructions.

The CRP measurements, performed prior to the scoring to select the suitable patients for the study, were run on the routinely used clinical chemistry analyser cobas c702 (Roche Diagnostics, Mannheim, Germany), according to the manufacturer’s original instructions.

Viral infections were detected using PCR analysis of respiratory samples. To investigate a broad spectrum of possible viral pathogens, various multiplex assays were utilised on different platforms, including the AllPlex RV Master Assay (Seegene, Seoul, South Korea), NeuMoDx 4-Plex Assay (Qiagen, Hilden, Germany), GeneXpert Xpress CoV-2/Flu/RSV Assay (Cepheid, Sunnyvale, USA), and cobas SARS-CoV-2 Assay (Roche, Rotkreuz, Switzerland). All assays were performed according to the respective manufacturers’ instructions. The decision regarding which viruses to test for was at the discretion of the physician in charge.

For blood culture diagnostics, 10–20 mL of blood were collected and inoculated into one or two aerobic and one or two anaerobic blood culture bottles (BacTec System; Becton Dickinson, Heidelberg, Germany). The cultures were incubated at 37 °C for 5 to 7 days, according to manufacturer’s instructions. Microbial identification was performed using biochemical testing systems (ATB, API, VITEK system; bioMérieux, Nurtingen, Germany) or matrix-associated laser desorption/ionisation time-of-flight (MALDI-TOF; Bruker Corporation, Billerica, MA, US).

### Ethics

This study adhered to the principles of the Declaration of Helsinki. The sample collection was performed in accordance with the International Conference on Harmonization - Good Clinical Practice and followed local regulatory requirements. In particular, the study protocol was approved by the Ethics Committee of the Technical University of Munich (approval no. 2022-370-S-SR). Written informed consent was obtained from all patients enrolled in this study.

### Statistical analysis

This was an explorative study, and no power analysis was performed. Data collection and calculations were performed using Microsoft Excel (Microsoft Corporation, Redmond, Washington, USA). Graphical depictions and statistical analyses were performed using R, version 4.03 (The R Foundation for Statistical Computing, Vienna, Austria). The distribution of continuous variables was described using medians and ranges. Categorical data were presented as absolute and relative frequencies. Owing to the inequality of variances, statistical significance was compared using Welch’s t-tests. A normal distribution was implied owing to the large sample size. *P* < 0.05 was considered statistically significant. The exclusion of outliers was discussed but rejected to represent real-life data.

## Results

### Baseline characteristics of the total cohort

In total, 97 patients were included in the study, and 31 patients were excluded (Fig. 1); forty-two (43.3%) were female and 55 (56.7%) were male. The median age was 66 years (interquartile range [IQR], 56–79 years), and 17 patients (17.5%) were admitted to the intensive care unit. Examining the comorbidities of participating patients, 58 (59.8%) had two or more comorbidities. Online Resources 1 and 2 present the underlying comorbidities for the viral and BSI groups. Eleven patients died during their inpatient stay.

### Baseline characteristics of patients with BSI

BSI was diagnosed in 56 patients (57.7%) (Fig. 1). In total, 19 different bacterial species and two *Candida* species were identified. The most common bacterial pathogens were *Escherichia coli (E. coli)* (18 patients, 32.1%) and *Klebsiella pneumoniae* (8 patients, 14.3%). Blood cultures were simultaneously positive for two bacterial species in seven patients. In three patients, blood cultures were simultaneously positive for three different bacterial species. Online Resource 3 presents the bacterial species detected.

The most common sources of BSI were urogenital and urinary (18 patients, 32.1%), abdominal (7 patients, 12.5%), and soft tissue (7 patients, 12.5%) infections. Ten patients (17.9%) developed neutropenic fever because of chemotherapeutic treatment for underlying haemato-oncological malignancies (Table 1). The median CRP level was 11.9 (interquartile range [IQR], 7.5–21.7) mg/dL; PCT, 2.4 (IQR, 0.6–13.3) ng/mL; and white blood cell count (WBC), 7.0 (IQR, 2.8–12.6) G/l. PCT values were collected at an interval of zero (IQR 0–1) days from the values of the host-protein signature score.

**Table 1:** Sources of bacterial infection in patients with blood stream infection

Source of infection, *n* (%)	Total (*N* = 56)
Urinary tract Neutropenic fever Abdominal Soft tissue Pulmonary Cholangitis Foreign body Spondylodiscitis Endocarditis Unclear source	18 (32.1) 10 (17.9) 7 (12.5) 7 (12.5) 5 (8.9) 5 (8.9) 5 (8.9) 1 (1.8) 1 (1.8) 1 (1.8)

n, number; N, total number of participants per group

Note: The parameters are displayed as absolute frequencies (relative frequency in %). The total number of detected sources of infection exceeded the total number of patients because some patients were diagnosed with more than one source of bacterial infection

All 56 patients with BSI received antibiotic treatment. Following our SOC, antibiotic therapy was initiated at a median of 1 day (IQR, 1–2 days) ahead of positive blood culture results because of suspected bacterial infection. Among the 56 patients with BSI, one did not receive antibiotic treatment before the blood culture became positive, as per SOC. This patient had a score of > 65 and would, therefore, have received antibiotic treatment earlier compared to the SOC.

The most common comorbidities were arterial hypertension (31 patients, 55.4%), diabetes mellitus (12 patients, 21.4%), atrial fibrillation (10 patients, 17.9%), and coronary artery disease (10 patients, 17.9%). Six patients (10.7%) received immunosuppressive therapy at the time of BSI. Two patients (3.6%) previously received an organ or stem cell transplantation, respectively (Online Resource 1).

### Results of the host-protein signature score and PCT in patients with BSI

**Fig. 2:**
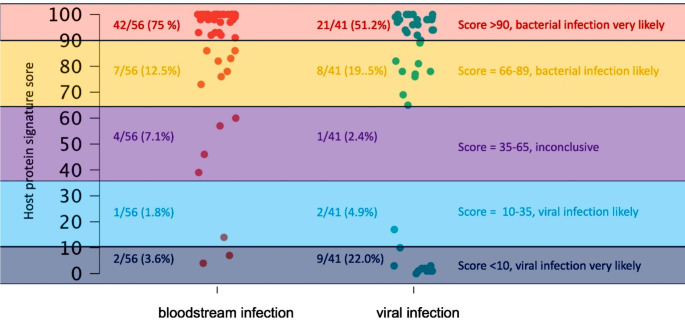
Graphical overview of the total cohort with host-protein signature score results

In patients with BSI (56 patients), the median host-protein score was 99 (IQR, 89.8–100). In total, 49 patients (87.5%) had a score > 65. In four patients (7.1%), the probability of a viral or bacterial infection was considered equivocal, with a score ranging from 36 to 65. Three patients (5.4%) with BSI had a score < 35, indicating the absence of a bacterial infection. (Fig. 2) The overall sensitivity for the score for identifying BSI was 87.5%. (Table 2) After excluding immunocompromised patients, sensitivity for the score for identifying BSI was 91.9%. (Table 3) PCT data were available for 47 patients (83.9%). The median PCT in patients with BSI was 2.4 ng/mL (IQR, 0.6–13.3 ng/mL). Using a cut-off of > 0.5 ng/mL, PCT demonstrated a sensitivity of 76.6% in detecting BSI. (Table 2). After excluding immunocompromised patients, sensitivity for PCT was 93.3%. (Table 3)

**Table 2:** Quality criteria of the host-protein signature score and PCT

Characteristic	Score > 65 (*N* = 78)	Score < 65 (*N* = 19)	PCT > 0.5 ng/mL (*N* = 48)	PCT < 0.5 ng/mL (*N* = 33)
BSI, n	49	7	36	11
Viral infection, n	29	12	12	22
Sensitivity, %	87.5		76.6	

N, total number of participants per group; PCT, procalcitonin; BSI, bloodstream infection; n, number

**Table 3:** Quality criteria of the host-protein signature score and PCT in immunocompetent patients

Characteristic	Score > 65 (*N* = 49)	Score < 65 (*N* = 10)	PCT > 0.5 ng/mL (*N* = 35)	PCT < 0.5 ng/mL (*N* = 14)
BSI, n	34	3	28	2
Viral infection, n	15	7	7	12
Sensitivity, %	91.9		93.3	

N, total number of participants per group; PCT, procalcitonin; BSI, bloodstream infection; n, number

Patients with immunosuppression, defined as patients receiving immunosuppressive therapy, neutropenic patients, solid-organ or stem cell transplanted patients, and patients with active lymphoma or leukaemia, were excluded

Among patients with a score > 65, 17 (34.7%) were tested positive for *E. coli*, 8 (16.3%) for *Klebsiella pneumoniae*, and six (12.2%) for *Staphylococcus aureus* (Online Resource 3). Immunosuppressive therapy was received by four patients (8.2%) (Online Resource 1). The median duration between initiation of antibiotic therapy and the score was 2 days (IQR, 1.0–3.0 days).

In the four patients with a score ranging from 35 to 65, the pathogens isolated from blood cultures were *Staphylococcus epidermidis*, *Enterococcus faecium*, *Streptococcus anginosus*, and *Proteus mirabilis* (Online Resource 3). One patient (25.0%) was receiving immunosuppressive therapy (Online Resource 1). The median duration between initiation of antibiotic therapy and the CLIA was 3.5 (IQR, 0.75–4) days. PCT data were available for two patients, with a median of 11.2 ng/mL.

In the three patients with a score < 35, the detected bacterial species were *Staphylococcus epidermidis*, *Staphylococcus hominis*, and *E. coli* (Online Resource 3). These were diagnosed in two cases of central catheter infections and urosepsis. One patient was receiving immunosuppressive therapy (Online Resource 1). All patients were treated with antibiotics, with a median time of 2 days before the CLIA. Data on PCT levels were available for two patients, with a median of 0.3 ng/mL.

### Baseline characteristics of patients with viral infections

A viral infection with negative blood culture results was diagnosed in 41 patients (Fig. 1). Severe acute respiratory syndrome coronavirus 2 (SARS-CoV-2) was the most commonly detected virus (24 patients, 58.5%). Influenza A/B was detected in 11 patients (26.8%) and respiratory syncytial virus (RSV) in seven patients (17.1%). One patient (2.4%) was diagnosed with human metapneumovirus, and one with dengue virus. Three patients (7.3%) were diagnosed with two viruses (Influenza A/RSV and Influenza A/SARS-CoV-2) simultaneously (Table 4). The median CRP was 8.6 (IQR, 6.1–14.5) mg/dL; PCT, 0.2 (0.1–1.2) ng/mL; and WBC 6.0 (IQR, 4.0–9.0) G/l. PCT values were collected at an interval of zero (IQR 0–1) days from the values of the host-protein signature score.

**Table 4:** Viral pathogens detected in patients with viral infections

Detected virus, *n* (%)	Total (*N* = 41)
SARS-CoV-2 Influenza A/B RSV Metapneumovirus Dengue	24 (58.5) 11 (26.8) 7 (17.1) 1 (2.4) 1 (2.4)

n, number; N, total number of participants per group; SARS-CoV-2, severe acute respiratory syndrome coronavirus 2; RSV, respiratory syncytial virus

Note: The parameters are displayed as absolute frequencies (relative frequency in %). The total number of detected viral pathogens exceeded the total number of patients because some patients were diagnosed with more than one viral pathogen

Following our SOC, 31 (75.6%) patients without BSI but with viral infections received antibiotic treatment, with a median of 1.5 days (1–2 days) before the CLIA. One patient (2.4%) received routinely administered antibiotic treatment after peroral endoscopic myotomy; there was no evidence of a bacterial infection. Table 5 illustrates the rationale for the antibiotic treatment. In 17 patients (53.1%), a pulmonary bacterial superinfection was suspected on diagnostic imaging, three patients had an additional bacterial urogenital focus (9.4%), three patients (9.4%) received antibiotic treatment due to neutropenic fever, two (6.3%) had a bacterial soft tissue infection, and one (3.1%) had a suspected cholangitis. In five patients with cancer (15.6%), calculated antibiotic therapy was administered owing to immunosuppression with distinctly increased infection markers and/or severe course of disease (low oxygen saturation/intubation) (Table 5).

**Table 5:** Reasoning behind antibiotic treatment in patients with primarily viral infections and without blood stream infection in relation to the host-protein signature score

Characteristic, *n* (%)	Antibiotic treatment (*N* = 32)	Score > 65 (*N* = 25)	Score 35–65 (*N* = 1)	Score < 35 (*N* = 6)
Respiratory bacterial superinfection Urocystitis Cholangitis Abdominal Endocarditis Soft tissue Spondylodiscitis Foreign body Neutropenic fever Calculated antibiotic therapy in patients with cancer	17 (53.1) 3 (9.4) 1 (3.1) 0 (0) 0 (0) 2 (6.3) 0 (0) 0 (0) 3 (9.4) 5 (15.6)	14 (56.0) 1 (4.0) 1 (4.0) 0 (0) 0 (0) 2 (8.0) 0 (0) 0 (0) 2 (8.0) 4 (16.0)	0 (0) 0 (0) 0 (0) 0 (0) 0 (0) 0 (0) 0 (0) 0 (0) 0 (0) 1 (100)	3 (50) 2 (33.3) 0 (0) 0 (0) 0 (0) 0 (0) 0 (0) 0 (0) 1 (16.7) 0 (0)

N, total number of participants per group; n, number

The parameters are displayed as absolute frequencies (relative frequency in %)

The most common comorbidities were arterial hypertension in 13 patients (31.7%) and atrial fibrillation in nine patients (22.0%). In total, 12 patients (29.3%) were receiving immunosuppressive therapy, and two patients (4.9%) were receiving B-cell depletion. Six patients (14.6%) underwent stem cell or organ transplantation (Online Resource 2).

### Host-protein signature score results in patients with viral infections

In total, 41 patients without BSI but with viral infections were included, and the median score was 90 (IQR, 13.5–98). Twenty-nine patients (70.7%) exhibited a score of > 65, while one patient (2.4%) had a score ranging from 35 to 65, and eleven patients (26.8%) had a score of < 35. (Fig. 2) Twenty-nine patients had a score > 65 and should have received antibiotic therapy according to the manufacturer’s recommendation. Of these 29 patients, 25 (86.2%) received antibiotic treatment following our SOC. Thus, four out of 29 patients with a score > 65 (13.8%) did not receive antimicrobial treatment, as no bacterial super/coinfection was suspected based on clinical assessment. These four patients, who were not treated with antibiotics following SOC, had respiratory infections: three cases of SARS-COV-2 and one of RSV. These patients’ mean PCT and CRP values were 0.87 ng/mL and 8.83 mg/dL, respectively. None of these patients died or required intensive care.

The patient with a score between 35 and 65 got antibiotic treatment due to an underlying oncological disease with immunosuppressive medication. In patients with a viral infection and a score < 35, SOC resulted in antibiotic treatment for suspected bacterial infection in six of eleven patients. In three of these six patients, a respiratory bacterial superinfection was suspected on diagnostic imaging; two patients were additionally diagnosed with a urogenital infection, and one had neutropenic fever. (Table 5) No patient died or required intensive care. PCT data were available for eight of eleven patients (72.7%), with a median of 0.1 (IQR, 0.1–0.4) ng/mL.

## Discussion

The accurate identification of bacterial and viral infections is essential to prevent the overuse of antibiotics. The host-protein signature score has primarily been tested in children with few comorbidities and viral infections [9–11]. This is the first time the efficacy and accuracy of the score was exclusively investigated in adult patients, many of whom suffered from several pre-existing conditions. Furthermore, the inclusion criteria encompassed all types of microbial infections, not solely respiratory infections or fever without an apparent source, as observed in most previous studies [12, 13].

In this prospective, explorative study, the score identified patients with bacteraemia with a high sensitivity of 87.5%. However, the sensitivity was slightly lower than that reported in a previous study involving adult patients, where a sensitivity of > 90% was reported [14]. Notably, 71.4% (35/49) of patients with a score > 65 exhibited gram-negative BSIs, while 85.7% (6/7) of those with a score ≤ 65 displayed gram-positive BSIs. One potential explanation for this phenomenon is that gram-negative bacteria elicit a more robust inflammatory response than do gram-positive bacteria. In particular lipopolysaccharides, located on the outer membrane of gram-negative bacteria, are potent immune stimulators that can activate the immune system [15, 16]. Another possible explanation could be that many of the gram-negative bacteria identified in the study are known to possess multidrug resistance. Hence, all patients were treated with broad-spectrum antibiotics, which also have a range of efficacy in gram-negative bacteria, which is why no sustained bacterial immune response in the gram-negative group and no subsequent higher score in this group was expected.

The performance of the score was compared with that of PCT, which is currently the laboratory marker of choice for better differentiation between bacterial and viral infections in our hospital. Identifying patients with BSI is crucial because BSIs are associated with higher mortality when antibiotic treatment is initiated inadequately or too late [17]. The score tended to demonstrate a higher sensitivity in detecting BSIs than that of PCT (87.5% vs. 76.6%) in this study, suggesting that combining multiple inflammation markers is more effective in identifying this vulnerable patient group. In comparison with SOC, one patient with a score > 65 did not receive antibiotic treatment prior to the blood culture becoming positive. Thus, antibiotic treatment could have been initiated earlier using the score.

AMS is playing an increasing role in Germany and is being promoted nationwide [18]. There is an S3 guideline that can be followed [19]. However, implementing the measures is the responsibility of the individual clinics. The hospital in which this study was conducted has an AMS staff unit that ensures the rational prescription of antibiotics. However, based on the clinical judgment of bacterial super/coinfection, the SOC is challenging and requires considerable expertise. Most patients with viral infections received antibiotic treatment, probably due to the high number of multimorbid patients, which sometimes requires deviations from the rather strict recommendations of the guidelines and in-house SOCs. In six patients with a viral infection, SOC resulted in antibiotic treatment for suspected bacterial infection, but the score was < 35, suggesting an overuse of antibiotic therapy in these patients. The positive impact of potential antibiotic savings is weakened as the score incorrectly identified four patients with viral infections, who were not receiving antibiotics by the SOC approach, as having bacterial infections. Thus, when compared to SOC, antibiotic therapy could have been avoided in only two cases with viral infections by using a host-protein signature score-guided approach, resulting in a minor effect. Whereby, as per the manufacturers’ recommendation, the score is not intended as a standalone diagnostic test for antimicrobial prescription. Considering the low number of cases, additional prospective studies are needed to further assess the accuracy and the benefits of a host-protein score guided approach in adults. Furthermore, economic considerations have not yet been made. Given the financial constraints faced by both hospital-based and outpatient practitioners, the host-protein-signature score and other tools (e.g., PCT) require a comparative cost-benefit analysis to determine its potential.

Our study has some limitations. First, the high rate of antibiotic treatments in patients with viral infections could be attributed to the study being conducted in a tertiary hospital, which tends to treat sicker and older patients with multiple pre-existing conditions and often bacterial and viral coinfections. A significant proportion of patients was immunocompromised, which might impair the immune response and, therefore, the calculation of the inflammation-marker-based CLIA-Score. A sub-analysis of immunocompetent patients revealed a higher sensitivity of the host-protein signature score, which supports this assumption. Further studies in both immunocompetent and immunocompromised patients are needed to understand the role of immunosuppression in host-marker scores and whether its reliability is dependent on the patient’s immune status.

Second, a gold standard for assessing the performance of the host-protein signature score was created by establishing strict inclusion criteria (CRP levels of > 5 mg/dL and fever, PCR-positive viral infection, or positive blood culture). However, this resulted in a lack of internal validity by prioritising hospitalised patients who were particularly ill. Furthermore, real-life scenarios could not be sufficiently imitated, as clinicians often face inconclusive clinical presentation and uncertainty of diagnostic tests [20]. Therefore, future prospective studies with a collective of adult patients are needed, where patients with multimorbidity and immunosuppression do not outnumber those with other diseases, and where real-life scenarios can be better represented, for instance by using a blinded expert committee to evaluate the disease aetiology. Third, PCT data were available for only 83.5% of patients. Finally, the results of the host-protein signature score may have been affected because antibiotic therapy was usually initiated before the CLIA.

In conclusion, in this explorative study, identifying patients with BSI tended to be more sensitive for the host-protein signature score than that for PCT. Nonetheless, with only two patients where antibiotic treatment could have been ceased, the overall impact of a host-protein signature score-guided approach to antibiotic treatment was minor when compared to that with the SOC. Given the limitations of this study and a low number of cases, further prospective investigations are needed to evaluate the efficacy of this score in adult patients, potentially with serial measurements of the host-protein signature score to gain a better discriminative ability. Economic factors should also be taken into consideration, by comparison of the implementation’s cost to its benefits.

## Electronic supplementary material

Below is the link to the electronic supplementary material.

Supplementary Material 1

## Data Availability

The data are available from the corresponding author upon request.
