# Supplementary material for: Evaluation of a host-protein signature score for differentiating between bacterial and viral infections: real-life evidence from a German tertiary hospital
Source: Infection. 2024 Sep 9;53(3):883–91. doi: 10.1007/s15010-024-02384-w (PMC12137434; doi:10.1007/s15010-024-02384-w)
Supplement: Supplementary file 1 — Supplementary Material 1 [file 15010_2024_2384_MOESM1_ESM.docx]

**SUPPLEMENTARY MATERIAL**

**Journal name:** ***Infection***

**Evaluation of a host-protein signature score for differentiating between bacterial and viral infections: Real-life evidence from a German tertiary hospital**

Laura Wagner^1^, Heike Schneider^2^, Peter B. Luppa^2^, Kathrin Schröder^3^, Nina Wantia^3^, Christiane Querbach^4^, Samuel D. Jeske^5^, Tobias Lahmer^1^, Kathrin Rothe^6^, Miriam Dibos^1^, Florian Voit^1^, Johanna Erber^1^, Christoph D. Spinner^1^, Jochen Schneider^1^, and Julian Triebelhorn^1^

1 TUM School of Medicine and Health, Department of Clinical Medicine – Clinical Department for Internal Medicine II, University Medical Center, Technical University of Munich, Munich, Germany

2 TUM School of Medicine and Health, Department of Clinical Chemistry and Pathobiochemistry, University Medical Center, Technical University of Munich, Munich, Germany

3 TUM School of Medicine and Health, Department of Medical Microbiology, Immunology and Hygiene, University Medical Center, Technical University of Munich, Munich, Germany

4 TUM School of Medicine and Health, Department of Pharmacy, University Medical Center, Technical University of Munich, Munich, Germany

5 TUM School of Medicine and Health, Department of Virology, University Medical Center, Technical University of Munich/ Helmholtz Centre Munich, Munich, Germany

**Corresponding author:**

Laura Wagner, MD

TUM School of Medicine and Health, Department of Clinical Medicine – Clinical Department for Internal Medicine II, University Medical Center, Technical University of Munich, Ismaninger Str. 22, 81675 Munich, Germany.

E-Mail: laura.wagner@mri.tum.de

**Online Resource 1: Comorbidities in patients with blood stream infection and depending on the results of the host-protein signature score.**

| Characteristic | BSI  (N=56) | Score > 65  (N=49) | Score  35-65  (N=4) | Score <35  (N=3) |
| --- | --- | --- | --- | --- |
| Cardiovascular disease, No. (%) | | | | |
| Myocardial Infarction  Chronic heart failure  Peripheral vascular disease  Arterial hypertension  Atrial fibrillation  Coronary Artery Disease | 3 (5.4)  9 (16.1)  3 (5.4)  31 (55.4)  10 (17.9)  10 (17.9) | 3 (6.1)  9 (18.4)  3 (6.1)  26 (53.1)  9 (18.4)  9 (18.4) | 0 (0)  0 (0)  0 (0)  3 (75.0)  1 (25.0)  1 (25.0) | 0 (0)  0 (0)  0 (0)  2 (66.6)  0 (0)  0 (0) |
| Pulmonary disease, No. (%) | | | | |
| COPD  Asthma  Chronical pulmonary disease | 1 (1.8)  1 (1.8)  0 (0) | 1 (2.0)  1 (2.0)  0 (0) | 0 (0)  0 (0)  0 (0) | 0 (0)  0 (0)  0 (0) |
| Renal disease, No. (%) | | | | |
| Acute kidney injury  Chronic kidney injury without dialysis  Chronic kidney injury with dialysis | 8 (14.3)  6 (10.7)  1 (1.8) | 8 (16.3)  6 (12.2)  0 (0) | 0 (0)  0 (0)  1 (25.0) | 0 (0)  0 (0)  0 (0) |
| Liver disease, No. (%) | | | | |
| Liver cirrhosis  Other chronic liver failure | 3 (5.4)  3 (5.4) | 3 (6.1)  3 (6.1) | 0 (0)  0 (0) | 0 (0)  0 (0) |
| Diabetes, No. (%) | | | | |
| Diabetes without damage  Diabetes with damage | 11 (19.6)  1 (1.8) | 9 (18.4)  0 (0) | 0 (0)  1 (25.0) | 2 (66.6)  0 (0) |
| Haemato-oncological disease, No. (%) | | | | |
| Leukaemia  Lymphoma  Other haemato-oncological disease | 6 (10.7)  4 (7.1)  4 (7.1) | 5 (10.2)  3 (6.1)  4 (8.2) | 1 (25.0)  0 (0)  0 (0) | 0 (0)  1 (33.3)  0 (0) |
| Solid tumour disease, No. (%) | | | | |
| Solid tumour, not metastasised  Solid tumour, metastasised | 9 (16.1)  5 (8.9) | 8 (16.3)  5 (10.2) | 1 (25.0)  0 (0) | 0 (0)  0 (0) |
| Immunosuppressive medication, No. (%) | | | | |
| B-cell depletion  Other immunosuppression | 0 (0)  6 (10.7) | 0 (0)  4 (8.2) | 0 (0)  1 (25.0) | 0 (0)  1 (33.3) |
| Transplant, No. (%) | | | | |
| Stem cell transplant  Organ transplant | 2 (3.6)  2 (3.6) | 2 (4.1)  1 (2.0) | 0 (0)  1 (25.0) | 0 (0)  0 (0) |
| Autoimmune disease, No. (%) | 2 (3.6) | 2 (4.1) | 0 (0) | 0 (0) |

The parameters are displayed as absolute frequencies (relative frequency in %). The total number of comorbidities exceeded the total number of patients because some patients were diagnosed with multiple comorbidities. BSI, blood stream infection; N, total number of participants per group; No., number; COPD, chronic obstructive pulmonary disease

**Online Resource 2: Comorbidities in patients with viral infections and depending on the results of the host-protein signature score.**

| Characteristic | Viral infection  (N=41) | Score  >65  (N=29) | Score  35-65 (N=1) | Score <35  (N= 11) |
| --- | --- | --- | --- | --- |
| Cardiovascular disease, No. (%) | | | | |
| Myocardial Infarction  Chronic heart failure  Peripheral vascular disease  Arterial hypertension  Atrial fibrillation  Coronary Artery Disease | 0 (0)  0 (0)  3 (7.3)  13 (31.7)  9 (22.0)  4 (9.8) | 0 (0)  0 (0)  2 (6.9)  10 (34.5)  8 (27.6)  4 (13.8) | 0 (0)  0 (0)  0 (0)  0 (0)  0 (0)  0 (0) | 0 (0)  0 (0)  1 (9.1)  3 (27.3)  1 (9.1)  0 (0) |
| Pulmonary disease, No. (%) | | | | |
| COPD  Asthma  Chronical pulmonary disease | 4 (9.8)  0 (0)  0 (0) | 1 (3.4)  0 (0)  0 (0) | 0 (0)  0 (0)  0 (0) | 3 (27.3)  0 (0)  0 (0) |
| Renal disease, No. (%) | | | | |
| Acute kidney injury  Chronic kidney injury  On dialysis | 8 (19.5)  4 (9.8)  1 (2.4) | 7 (24.1)  4 (13.8)  0 (0) | 0 (0)  0 (0)  0 (0) | 1 (9.1)  0 (0)  1 (9.1) |
| Liver disease, No. (%) | | | | |
| Liver cirrhosis  Other chronic liver failure | 1 (2.4)  1 (2.4) | 1 (3.4)  1 (3.4) | 0 (0)  0 (0) | 0 (0)  0 (0) |
| Diabetes, No. (%) | | | | |
| Diabetes without damage  Diabetes with damage | 2 (4.9)  1 (2.4) | 2 (6.9)  1 (3.4) | 0 (0)  0 (0) | 0 (0)  0 (0) |
| Haemato-oncological disease, No. (%) | | | | |
| Leukaemia  Lymphoma  Other haemato-oncological disease | 6 (14.6)  8 (19.5)  1 (2.4) | 5 (17.2)  4 (13.8)  1 (3.4) | 1 (100)  0 (0)  0 (0) | 0 (0)  4 (36.4)  0 (0) |
| Solid tumour disease, No. (%) | | | | |
| Solid tumour, not metastasised  Solid tumour, metastasised | 3 (7.3)  7 (17.1) | 3 (10.3)  4 (13.8) | 0 (0)  0 (0) | 0 (0)  3 (27.3) |
| Immunosuppressive medication, No. (%) | | | | |
| B-cell depletion  Other immunosuppression | 2 (4.9)  12 (29.3) | 2 (6.9)  8 (27.6) | 0 (0)  1 (100) | 0 (0)  3 (27.3) |
| Transplant, No. (%) | | | | |
| Stem cell transplant  Organ transplant | 6 (14.6)  6 (14.6) | 5 (17.2)  5 (17.2) | 0 (0)  0 (0) | 1 (9.1)  1 (9.1) |
| Autoimmune disease, No (%) | 0 (0) | 0 (0) | 0 (0) | 0 (0) |

The parameters are displayed as absolute frequencies (relative frequency in %). The total number of comorbidities exceeded the total number of patients because some patients were diagnosed with multiple comorbidities. N, total number of participants per group; No., number; COPD, chronic obstructive pulmonary disease.

**Online Resource 3: Bacterial species in patients with blood stream infection.**

| Bacterial species | Total  (N= 56) | Score >65  (N= 49) | Score 65-35  (N=4) | Score <35  (N=3) |
| --- | --- | --- | --- | --- |
| Gram-positive, No. (%) | | | | |
| *Staphylococcus epidermidis*  *Staphylococcus aureus*  *Enterococcus faecalis*  *Enterococcus faecium*  *Streptococcus anginosus*  *Staphylococcus haemolyticus*  *Staphylococcus hominis*  *Streptococcus pyogenes*  *Streptococcus vestibularis*  *Streptococcus oralis*  *Clostridium perfringens* | 6 (10.7)  6 (10.7)  4 (7.1)  4 (7.1)  2 (3.6)  3 (5.4)  1 (1.8)  1 (1.8)  1 (1.8)  1 (1.8)  1 (1.8) | 3 (6.1)  6 (12.2)  4 (8.2)  3 (6.1)  1 (2.0)  3 (6.1)  0 (0)  1 (2.0)  1 (2.0)  1 (2.0)  1 (2.0) | 2 (50.0)  0 (0)  0 (0)  1 (25.0)  1 (25.0)  0 (0)  0 (0)  0 (0)  0 (0)  0 (0)  0 (0) | 1 (33.3)  0 (0)  0 (0)  0 (0)  0 (0)  0 (0)  1 (33.3)  0 (0)  0 (0)  0 (0)  0 (0) |
| Gram-negative, No. (%) | | | | |
| *Escherichia coli*  *Klebsiella pneumoniae*  *Proteus mirabilis*  *Pseudomonas aeruginosa*  *Enterobacter cloacae*  *Morganella morganii*  *Phocaeicola vulgatus*  *Serratia marcescens* | 18 (32.1)  8 (14.3)  3 (5.4)  2 (3.6)  3 (5.4)  1 (1.8)  1 (1.8)  1 (1.8) | 17 (34.7)  8 (16.3)  2 (4.1)  2 (4.1)  3 (6.1)  1 (2.0)  1 (2.0)  1 (2.0) | 0 (0)  0 (0)  1 (25.0)  0 (0)  0 (0)  0 (0)  0 (0)  0 (0) | 1 (33.3)  0 (0)  0 (0)  0 (0)  0 (0)  0 (0)  0 (0)  0 (0) |
| Candida, No. (%) | | | | |
| *Candida parapsilosis*  *Candida krusei* | 1 (1.8)  1 (1.8) | 1 (2.0)  1 (2.0) | 0 (0)  0 (0) | 0 (0)  0 (0) |

The parameters are displayed as absolute frequencies (relative frequency in %). The total number of detected bacterial species exceeded the total number of patients because multiple bacterial species were detected in some patients. N, total number of participants per group; No., number.
